# The effect of fetal gender on the biochemical markers of the first-trimester screening

**DOI:** 10.15537/smj.2022.43.4.20210906

**Published:** 2022-04

**Authors:** Hakan Cokmez, Simge Tezel Yozgat

**Affiliations:** *From the Department of Obstetrics and Gynecology (Cokmez), Izmir Ataturk Training and Research Hospital, Izmir; and from the Department of Obstetrics and Gynecology (Tezel Yozgat), Balikesir Ataturk City Hospital, Balikesir, Turkey.*

**Keywords:** chorionic gonadotropin, pregnancy-related plasma protein A, fetal gender

## Abstract

**Objectives:**

To determine the effects of fetal gender on the maternal levels of first-trimester screening biochemical markers, such pregnancy-related plasma protein A (PAPP-A) and beta-human chorionic gonadotropin (β-hCG).

**Methods:**

In this retrospective study, we assessed 267 cases of singleton pregnancies, who underwent first trimester screening tests and delivered between January 2016 and January 2019 at our hospital. Multiple of median (MoM) levels of PAPP-A and free β-hCG, and the neonatal genders according to the birth records were compared and analyzed. Additionally, patients with small for gestational age (SGA) newborns, preeclampsia, and placental ablation, called ischemic placental diseases, were classified into a separate group and their PAPP-A and free β-hCG MoM values and fetal genders were compared.

**Results:**

There was no significant relationship between the mean values of PAPP-A (1.07±0.6) and free β-hCG (1.23±1.14) and the fetal gender (males: 137, 51.3%; females: 130, 48.7%), respectively (*p*=0.833; *p*=0.075). In 41 cases (15.4%) with ischemic placental disease, free β-hCG values was significantly higher in the fetal females (19 cases; 46.3%) than males (22 cases; 53.7%), (1.53±1.02 and 0.77±0.53, respectively), (*p*=0.004).

**Conclusion:**

Pregnancy-related plasma protein A and free β-hCG values were not affected by the fetal gender. However, the significant relationship observed between free β-hCG MoM levels and fetal gender in patients with ischemic placental diseases suggests the need for larger studies on this topic.

**C**urrently, first-trimester screening tests are routinely recommended for every pregnant woman between the 11th and 14th gestational week to determine the risk of trisomy 15, 18, and 21. The biochemical markers of the first trimester screening test are pregnancy-related plasma protein A (PAPP-A) and beta-human chorionic gonadotropin (β-hCG). Pregnancy-related plasma protein A is a metalloproteinase whose concentration increases due to the expansion of the placenta at the end of the first trimester, breaks down insulin-like growth factor-binding proteins, and is synthesized by the trophoblasts.^1,2^ Beta-human chorionic gonadotropin is a hormone with a glycoprotein structure and is synthesized in the placenta by the syncytiotrophoblasts^3^ and ensures continued progesterone synthesis from the corpus luteum by binding to luteinizing hormone receptors in the first trimester of pregnancy.

Multiple studies that investigated the relationship between poor pregnancy results and biochemical markers of the screening test reported that maternal blood levels of PAPP-A and β-hCG can be used for early detection of placental complications that may occur in the succeeding weeks as well as fetal trisomies.^4-7^ Since both PAPP-A and β-hCG are synthesized in the placenta, they are common products of maternal and fetal cells. The role of the fetus in placental development raises the question of whether PAPP-A and β-hCG levels are affected by the fetal gender in ischemic placental diseases, which include mall for gestational age (SGA) newborn, hypertension above 140/90 mmHg that was not present before the pregnancy, and non-traumatic placental ablation.^8-10^ Some studies have demonstrated that both perinatal morbidity and mortality and maternal PAPP-A and β-hCG levels are affected by the fetal gender.^11-14^ Additionally, other studies have reported that preeclampsia and primary cesarean section rates in pregnant women with male fetuses are higher compared with pregnant women with female fetuses.^15,16^

The aim of this study was to investigate the effects of fetal gender on maternal serum PAPP-A and free β-hCG levels.

## Methods

In this retrospective cohort study, 267 cases who underwent delivery between January 2016 and January 2019 in a single tertiary center were included. The study was carried out in accordance with the 1964 Helsinki Declaration, revised in 2013. Due to the retrospective design of the study and the anonymous data used in the analyses, informed consent was not obtained from the patients. Ethics committee approval was obtained from our institutional committee before initiating the study (#475/2019).

Data regarding the demographics, pregnancy results, neonatal gender, and ultiple of median (MoM) levels of the biochemical parameters were obtained from the hospital records. During the study period, PAPP-A and free β-hCG levels were assessed using Siemens IMMULITE® 2000 XPi immunoassay system (Siemens Healthcare GmbH, Erlangen, Federal Republic of Germany). For MoM calculations, Siemens PRISCA prenatal risk calculation system was used during the study period. Singleton pregnancies in which both the first trimester screening test and delivery (gestation >24 weeks) were performed at our institution in adult women of >18 and <39 years of age were included in the study. Adolescents and pregnant women with advanced age (>39 years) were excluded due to their higher risk of obstetric complications because age could be a confounding factor. Additionally, pregnant women with chronic diseases (type 2 diabetes and autoimmune and chronic cardio-vascular diseases), pregnancies that had required assisted reproductive techniques, and pregnant women who were smokers were also excluded.

The subjects included in the study were classified according to the neonatal gender and were compared using the calculated mean values of PAPP-A and free β-hCG MoM levels in the first trimester screening test. Additionally, the calculated mean values of PAPP-A and free β-hCG MoM levels in pregnant women with ischemic placental diseases were also compared with the neonatal gender. The gestational age was calculated based on the fetal head-breech length measured on ultrasound in the first trimester.

Additionally, the calculated mean values of PAPP-A and free β-hCG MoM levels in pregnant women with ischemic placental diseases were also compared with the neonatal gender. Preeclampsia, SGA, and non-traumatic ablation are obstetrical complications about the placenta. Uteroplacental ischemia may be a factor that is responsible for these 3 conditions. These conditions are called ischemic placental disease as described in the literature.^10^ In this study, preeclampsia was defined as systolic blood pressure of 140 mm Hg or more or diastolic blood pressure of 90 mm Hg or more after 20 weeks of gestation with proteinuria (protein/creatinine ratio of 0.3 mg/dL or more or dipstick reading of 2+), SGA was defined as birth weight below the 10th percentile of the birth-weight-for-gestational-age reference curve, ablation was determined as vaginal bleeding, uterine tenderness, fetal distress and accompanied by postpartum retroplacental clots on the placenta.^10^

Continuous numerical data were expressed as mean ± standard deviation, while intermittent numerical data were expressed as numbers, and nominal data were expressed as numbers and percentages. The distribution of continuous data was determined using Kolmogorov–Smirnov test. In the comparative analysis of continuous data, student’s t-test was used in cases of normal distribution, and Mann–Whitney U test was used in cases of non-normal distribution. All analyses were performed using SPSS version 20.0 (IBM Inc., Armonk, NY, USA). Results with *p*<0.05 were considered statistically significant.

## Results

Overall, 267 patients with a mean age of 28.5±6.1 years were included. Of them, 59 (22.1%) were nulliparous and 208 (77.9%) were multiparous. Additionally, 61 (22.8%) patients had past history of at least one abortion. The pregnancies included in the study ended by cesarean section in 122 (45.7%) patients and vaginal delivery in 145 (54.3%) patients.

Stillbirth was observed in 2 (0.7%) cases. Post-delivery, 137 (51.3%) neonates were males and 130 (48.7%) were females. The average birth weight of the neonates was 3359.2±406.3 grams. The mean PAPP-A MoM level was 1.07±0.6; the mean free β-hCG MoM level was 1.23±1.14. The comparisons of serum PAPP-A and free β-hCG MoM levels and birth weights according to the neonatal gender are summarized in Table 1.

**Table 1:** - Comparisons of serum PAPP-A and free β-hCG levels and birth weight according to the neonatal gender.

Data*	Female	Male	*P*-value
PAPP-A (MoM)	1.07±0.63	1.07±0.62	0.833^†^
Free β-hCG (MoM)	1.35±2.07	1.12±1.08	0.075^†^
Birth weight, grams	3244.8±417.4	3372.5±386.7	0.010^‡^

*Mean ± standard deviation. ^†^Mann–Whitney U test. ^‡^T-Test. PAPP-A: pregnancy-related plasma protein A, β-hCG: free beta-human chorionic gonadotropin, MoM: multiple of median

Overall, 41 (15.4%) patients had encountered at least one of ischemic placental diseases during their pregnancy (Table 2). The comparisons of serum levels of PAPP-A and free β-hCG MoM in these cases according to the neonatal genders are summarized in Table 3. In addition, the comparison of patients with or without placental ischemic disease according to the neonatal genders are presented in Table 4.

**Table 2:** - Distribution of ischemic placental diseases

Ischemic placental disease	Number of cases* (n)
SGA newborn	8
Pregnancy related hypertension	35
Non-traumatic placental ablation	4

SGA: Small for gestational age. *Also includes cases with more than one ischemic placental disease.

**Table 3:** - Comparisons of serum levels of PAPP-A and free β-hCG in pregnant women with ischemic placental disease according to neonatal gender.

Data*	Female	Male	*P*-value^¥^
PAPP-A (MoM)	1.16±0.83	1.09±0.87	0.803
Free β-hCG (MoM)	1.53±1.02	0.77±0.53	0.004

*Mean±standard deviation. ^¥^Mann-Whitney U test. PAPP-A: pregnancy-related plasma protein A, β-hCG: free beta-human chorionic gonadotropin, MoM: multiple of median

**Table 4:** - Comparison of patients with or without placental ischemic disease according to the neonatal genders.

Data*	Male	Female	*P*-value^†^
Cases without IPD	115 (50.9)	111 (49.1)	0.744
Cases with IPD	22 (53.7)	19 (46.3)	

*n(%). ^†^Pearson Chi-square, IPD: Ischemic placental disease

## Discussion

There was no significant relationship between the mean values of PAPP-A and free β-hCG and the fetal gender. The most striking observation in our study was that in patients who encountered at least one of the ischemic placental diseases during the pregnancy, the mean MoM level of free β-hCG was significantly different between the female and male fetal genders. We also investigated the effect of fetal gender on the relationship between ischemic placental diseases and the first trimester screening biochemical parameters PAPP-A and free β-hCG.

Spencer et al,^17^ in their investigation of the changes in first-trimester screening parameters according to fetal gender in 2923 pregnant women, reported that PAPP-A and free β-hCG MoM levels were higher by 10-15% in women with female fetuses compared with those with male fetuses. Hence, they stated that this may result in a 1-2% change in the detection of trisomy 21 detection in female fetuses. Cowans et al,^18^ in their investigation of the changes in first trimester screening parameters according to fetal gender in 56,024 pregnant women, reported that the mean free β-hCG MoM level was 14.7% higher in women with female fetuses compared with those with male fetuses. Both studies stated the uncertainty in correcting the values of the first trimester screening test according to fetal gender and their results on the clinical efficacy since the determination of fetal gender is difficult in the first trimester.^17,18^ Ischemic placental diseases mostly occur in the last trimester and the fetal gender can be detected before this trimester more easily. Therefore, the determined fetal gender before the third trimester and first trimester biochemical parameters may be used for predicting the risk of ischemic placental disease. However, there is no cut-off value for these biochemical parameters in the literature yet. Thus, in terms of predicting the risk of ischemic placental disease, studies should be carried out to identify a cut-off value, determined according to the fetal gender, for first trimester biochemical parameters. In studies that investigated changes in first trimester screening test parameters according to fetal gender, some reported that free β-hCG MoM level and PAPP-A MoM level were higher in female fetuses.^13,18-20^

In our study, we did not find a significant relationship between both PAPP-A and free β-hCG MoM levels and the fetal gender. However, in the group of patients with at least one of the ischemic placental diseases, we found the free β-hCG MoM levels to be higher in women with female fetuses than those with male fetuses. Intrauterine growth retardation is more common in female fetuses.^21^ In some studies that did not include fetal gender classification, high free β-hCG MoM level in the first trimester screening test was found to be associated with low birth weight according to the week of gestation and placental ablation.^6,7^

In their study Khalil and Alzahra^15^ investigated the association of poor pregnancy outcomes and male-bearing gravids in 29,140 patients, they confirmed the effect of a male fetus on the existence of preeclampsia in their study population. In our study, we did not find a significant relationship between fetal gender and ischemic placental disease.

### Study limitations

A limitation of this study is the loss of data because the first trimester screening test reports before 2017 were not uploaded to the system. Furthermore, our neonatal intensive care unit was closed during the study period, which resulted in a mismatch between the number of outpatient patients and the number of deliveries. Another limitation is the limited number of patients included in the study. Additionally, the single-centered and retrospective design includes inherent weaknesses of the design. The distribution of ischemic placental disease in our study was not suitable for statistical analysis. In addition, this study has included cases with more than one ischemic placental disease. Multicenter studies with large sample sizes on patients with placental ischemic disease may overcome this problem.

The strength of our study is that it investigates the effect of fetal gender on the first trimester screening biochemical parameters PAPP-A and β-hCG in the context of ischemic placental diseases.

In conclusion, we found that, in patients who suffer at least one of the ischemic placental diseases during pregnancy, the mean MoM levels of free β-hCG were significantly different between female and male fetuses. Therefore, we believe that further studies are required to investigate the relationship between fetal gender and free β-hCG MoM levels to contribute to the understanding of the pathogenesis and to determine a cut-off value according to fetal gender in ischemic placental diseases.
